# Sex Specification and Heterogeneity of Primordial Germ Cells in Mice

**DOI:** 10.1371/journal.pone.0144836

**Published:** 2015-12-23

**Authors:** Akihiko Sakashita, Yukiko Kawabata, Yuko Jincho, Shiun Tajima, Soichiro Kumamoto, Hisato Kobayashi, Yasuhisa Matsui, Tomohiro Kono

**Affiliations:** 1 Department of Bioscience, Tokyo University of Agriculture, Setagaya, Tokyo, Japan; 2 NODAI Genome Research Center, Tokyo University of Agriculture, Setagaya, Tokyo, Japan; 3 Cell Resource Center for Biomedical Research, Institute of Development, Aging and Cancer, Tohoku University, Sendai, Miyagi, Japan; National University of Singapore, SINGAPORE

## Abstract

In mice, primordial germ cells migrate into the genital ridges by embryonic day 13.5 (E13.5), where they are then subjected to a sex-specific fate with female and male primordial germ cells undergoing mitotic arrest and meiosis, respectively. However, the sex-specific basis of primordial germ cell differentiation is poorly understood. The aim of this study was to investigate the sex-specific features of mouse primordial germ cells. We performed RNA-sequencing (seq) of E13.5 female and male mouse primordial germ cells using next-generation sequencing. We identified 651 and 428 differentially expressed transcripts (>2-fold, P < 0.05) in female and male primordial germ cells, respectively. Of these, many transcription factors were identified. Gene ontology and network analysis revealed differing functions of the identified female- and male-specific genes that were associated with primordial germ cell acquisition of sex-specific properties required for differentiation into germ cells. Furthermore, DNA methylation and ChIP-seq analysis of histone modifications showed that hypomethylated gene promoter regions were bound with H3K4me3 and H3K27me3. Our global transcriptome data showed that in mice, primordial germ cells are decisively assigned to a sex-specific differentiation program by E13.5, which is necessary for the development of vital germ cells.

## Introduction

Oocytes and spermatozoa are derived from foetal primordial germ cells (PGCs), which appear as a small cell population on embryonic day 7.25 (E7.25). In mice, progenitors are identified by expression of *Blimp1*/*Mil1*, which is localized to the extra-embryonic mesoderm of the posterior amniotic fold [1–3]. PGCs proliferate and migrate to the genital ridges that form on the ventral surface of the mesonephros as paired thickenings of the epithelial layers [4, 5]. The genital ridges remain bipotential until transient sex-determining region Y (*Sry*) expression from the Y chromosome occurs in the genital ridges of E11.0–12.0 male mice. Thus, the fate of bipotential gonads is specified by E13.5, resulting in subsequent differentiation into either ovaries or testes [6, 7].

Once PGCs complete the migration to the genital ridge, their sex-specific fate is determined, and the differentiation programs of female and male germ cells are initiated [8–10]. However, the underlying mechanisms of sex-specific fate determination are not fully elucidated. A previous transcriptome study using microarray analysis showed that sexually undifferentiated PGCs were primed with a bias towards a male fate, whereas supporting cell progenitors were primed towards a female fate by E13.5 [11]. Moreover, we identified gene sets exclusively expressed in female and male PGCs, thus implicated them in sex-specific fate determination.

After global demethylation, which occurs until mid-gestation (E13.5) [12], female and male PGCs undergo sex-specific fate determination. Briefly, male PGCs enter mitotic arrest, whereas female PGCs immediately enter prophase of meiosis I [10, 13]. To date, an important question regarding the uniformity of PGCs has not been addressed. It is known that the number of PGCs peaks at this stage, after which the cells enter an attrition stage and their numbers decease via apoptosis [14–16]. The molecular mechanisms underlying the initiation of distinct PGC-differentiation programs are not fully elucidated. Global transcriptome analysis and bioinformatics of PGCs could shed insight into these complex processes. Furthermore, it is important to determine whether these cells are capable of self-determining their own survival or death. Exploring these key questions should provide a better understanding of mammalian reproductive processes.

Epigenetic regulation of gene expression is involved in germline differentiation and PGC sex specification. Previously, we reported the global and single-resolution DNA methylome map of germline cells using a post-bisulphite, adapter-tagging method. We showed that DNA is globally hypomethylated in both female and male PGCs by E13.5. After global demethylation, which lasts until mid-gestation (E13.5) [12], PGCs undergo distinct fate determinations. RNA-sequencing (seq) and ChIP-seq analyses of female and male PGCs during sex specification and female meiotic initiation were previously performed to study the regulation of gene expression via histone modifications [17–20]. These results showed that a subset of genes in PGCs was maintained in a bivalent state of H3K4me3 and H3K27me3. These particular histone modifications were also demonstrated in embryonic stem cells (ESCs) [21, 22].

Thus, we sought to determine whether complex networks regulating gene expression levels are involved in PGC sex specification. We conducted a transcriptome analysis by performing deep RNA-seq of E13.5 mouse PGCs. A ChIP-seq analysis of H3K4me3 and H3K27me3 was also performed to evaluate the epigenetic regulation of PGCs. In this study, we successfully identified female and male-specific PGC-expressed gene sets and demonstrated complex gene expression networks that are implicated in sex specification, leading to female and male PGC differentiation.

## Materials and Methods

### Ethics statement

This study was carried out in strict accordance with the Tokyo University of Agriculture Guide for Care and Use of Laboratory Animals. The protocol was approved by the Committee on the Ethics of Animal Experiments of the Tokyo University of Agriculture (Permit Number: 260064SE). At the time of sample collection, all animals were sacrificed by cervical dislocation, and all efforts were made to minimize suffering.

### Animals and PGC collection

Female C57BL/6 mice, 10–12 weeks of age, (Clea Japan, Tokyo, Japan) were crossed with male homozygous *Pou5f1-ΔPE-GFP* mice (C57BL/6 background) [23]. The day that the vaginal plug was first detected was defined as E0.5. Heterozygous *Pou5f1-ΔPE-GFP* embryos were recovered at E13.5, and the sex was distinguished based on the morphology of the genital ridge. The genital ridge was removed and treated with a 1 mg/ml collagenase solution (Wako) at 37°C for 40 min, followed by treatment with 0.25% trypsin-EDTA solution (0.53 mM; Sigma) at 37°C for 15 min. After adding foetal bovine serum (FBS), a single-cell suspension was obtained by gentle pipetting. GFP-positive cells (PGCs) were isolated and collected using a FACSAria II cell sorter (BD Biosciences; S1A Fig).

### Immunofluorescence analysis for the determination of PGC purity

The collected PGCs were resuspended in M2 medium and incubated with a PE-conjugated mouse anti-mouse SSEA1 monoclonal antibody (560142, BD Pharmingen: 1/25 dilution) at 4°C for 1 h. After antibody incubation, cells were mounted on glass slides and visualized using an LSM710 laser-scanning confocal microscope (Zeiss). The recovered cells comprised an SSEA1-positive cell population (>97%; S1B Fig).

### ESC derivation and culture

C57BL/6 male mouse ESCs were co-cultured with inactivated mouse embryonic fibroblasts (MEFs) in ESC medium (15% KSR, 0.055 mM β-mercaptoethanol, 2 mM L-glutamine, 0.1 mM MEM non-essential amino acids, and 5000 u/ml penicillin/streptomycin in Knockout DMEM) with 2i (PD0325901, 0.4 μM; Stemgent, San Diego, CA; CHIR99021, 3 μM Stemgent) and LIF (1000 u/ml; Chemicon). The expanded ESC colonies were collected following dissociating with 0.25% trypsin-EDTA (0.53 mM) solution.

### RNA isolation and RNA-seq library preparation

Total RNA from PGCs and ESCs (1 × 10^4^ cells) was isolated using an RNeasy Micro Kit (QIAGEN), with DNase treatment. DNA synthesis and pre-amplification were performed with total RNA (10 ng) using a SMARTer Ultra Low Input RNA Kit and an Advantage 2 PCR Kit (Clontech USA), respectively, according to the manufacturers’ instructions. Pre-amplified cDNA was fragmented into 200-bp fragments using an S2 sonicator (Covaris, USA) and then used to construct sequencing libraries using a NEBNext Ultra DNA Library Prep Kit, following the manufacturers’ protocol (New England BioLabs). Indexed libraries were pooled (10 nM each), and sequenced using an Illumina MiSeq sequencer (single-end, 150 bp condition). Two biological replicates were used for each sample.

### RNA-sequence alignments and statistical analysis

RNA-seq reads for each sample were aligned to the mouse genome (mm10, Genome Reference Consortium Mouse Build 38) with the CLC Genomics Workbench (CLC Bio). Aligned reads were subsequently assembled into transcripts guided by reference annotation (mm10, UCSC gene annotation). Transcript expression was quantified in terms of reads per million mapped reads and normalized using the trimmed mean of M values method with Strand NGS (Agilent).

### Single PGC capturing and cDNA synthesis

Single E13.5 PGCs were captured on a medium-sized (10–17-μm cell diameter) integrated fluidic circuit (IFC, Fluidigm). Cells were loaded onto the IFC chip at a concentration of 1000 cells/μl, simultaneously stained for cell viability using a LIVE/DEAD Cell Viability/Cytotoxicity Kit (Invitrogen), and observed by fluorescence microscopy to assess the number and viability of cells per capture site. For single PGC RNA-seq, cDNA synthesis and pre-amplification were performed on an IFC chip using the C1 Single-Cell Auto Prep System method (Fludigm) with a SMARTer Ultra Low Input RNA Kit and Advantage 2 PCR Kit (Clontech USA), respectively.

### Identification of sex-specific expressed genes

To visualize the gene expression patterns for each RNA-seq dataset, we generated scatter plots for each sample, using the R psych package (http://personality-project.org/r/psych/). Two biological-replicate samples were combined, and the average expression level of each sample was computed using Strand NGS (Agilent). To identify significant differentially expressed genes between female and male PGCs, we used 2 selection criteria. The first criterion was a fold-change in expression of at least two-fold. The second criterion was removal of false positive genes using a moderated *t* test with Benjamini—Hochberg false-discovery rate (FDR) of <0.05). Using this approach, we extracted 651 FSGs and 428 MSGs.

### Functional enrichment analysis with gene ontogeny (GO) and pathway analysis

FSG and MSG lists were used for GO analysis with the DAVID web tool (http://david.abcc.ncifcrf.gov/) [24]; a background of all mouse genes was applied. Biological-process term groups with a significance of *P* < 0.01 (modified Fisher’s exact test) were considered significant. All transcript lists of female and male PGCs were studied via pathway analysis using DAVID (http://david.abcc.ncifcrf.gov/) [24], with a background of all mouse genes. GO term groups with a significance of *P* < 0.01 (modified Fisher`s exact test) were considered significant. Visual biological pathway maps were generated using a BioCarta annotation source.

### Detection of expression alleles

Total RNA obtained from PGCs of E13.5 BDF1 (C57B6 × DBA) foetuses was used for cDNA synthesis. The expressed allele of imprinted genes was detected by SNPs from the sequencing results of RT-PCR products. Primers sequences and PCR conditions are shown in S4 Table.

### Chromatin immunoprecipitation (ChIP) assays and ChIP-seq library preparation

Chromatin preparation for ChIP assays was performed using a SimpleChIP Plus Enzymatic Chromatin IP Kit (Cell Signaling Technology), according to the manufacturer’s protocol. For each chromatin preparation, 1 × 10^5^ PGCs were cross-linked for 2 min using 1% formaldehyde. After cell lysis, chromatin was digested by micrococcal nuclease for 20 min at 37°C and was fragmented by sonication using a BioRuptor sonicator (Diagenode UCD-200) set to high power for 20 min, with alternating ‘ON’ (30 s) and ‘OFF’ (30 s) sequences. ChIP assays were performed with 2 μg of rabbit anti-H3K4me3 polyclonal antibody (39915, Active Motif) and 4 μg of rabbit anti-H3K27me3 polyclonal antibody (39155, Active Motif) antibodies conjugated with Dynabeads Protein A (Life Technologies). The chromatin solution was diluted with ChIP dilution buffer (50 mM Tris-HCl, 167 mM NaCl, 1.1% Triton X-100, and 0.11% sodium deoxycholate). Ten percent of the diluted chromatin sample was used as an input. Diluted chromatin samples were incubated with antibody/bead complexes overnight at 4°C with rotation. Chromatin/antibody/bead complexes were washed according to the instructions provided with the LowCell# ChIP Kit (Diagenode), eluted from the beads by the addition of 200 μl of elution buffer, and incubated overnight at 65°C. Next, the complexes were digested with RNase A for 30 min at 37°C and proteinase K for 2 h at 55°C. Input samples were reverse cross-linked at this stage, DNA was subsequently purified using AMpure beads, and sequencing libraries were constructed using the NEXTflex^™^ ChIP-Seq Kit and NEXTflex^™^ DNA Barcodes (BIOO Scientific Corp.). Indexed libraries were pooled (10 nM each), and sequenced using an Illumina HiSeq 2500 sequencer (single-end, 50-bp condition). Two biological replicates were used for each sample.

### ChIP-seq alignment and statistical analysis

ChIP-seq reads were aligned to the mouse genome (mm10, Genome Reference Consortium Mouse Build 38) using bowtie2-2.2.3 with the default settings (http://bowtie-bio.sourceforge.net/bowtie2/index.shtml). Only unique hits were used for further analysis. ChIP/input enrichment-peak regions were calculated using the DROMPA2.6.4 peak-calling program with default parameters (http://www.iam.u-tokyo.ac.jp/chromosomeinformatics/rnakato/drompa/) [25]. To evaluate histone modifications of FSGs and MSGs, the number of reads in the transcription start site (TSS) and transcription termination site (TTS) shore region (±5 kb of the TSS and TTS) was determined. First, each aligned read was converted to one base sequence at the 85^th^ base position from the read starting point, considered as the center of the fragment, and the number of reads was counted for each non-overlapping 100-bp bin. The count was adjusted relative to the total read count. Composite profiles were generated using the SeqMonk program (http://www.bioinformatics.babraham.ac.uk/projects/seqmonk/).

### Methylation analysis around the TSSs of expressed sex-specific genes

The TSS shore (±5 kb of TSS) regions in FSGs and MSGs were divided into 100-bp bins. For each bin, the average methylation level was calculated for each gene, using our previously obtained E13.5 female and male methylome dataset (Accession Number DRA000607) [12].

### Principal component analysis (PCA) and clustering analysis

Single-cell transcriptome datasets were applied to PCA, hierarchical cluster analysis, and k-means cluster analysis. The gene lists of single RNA-seq datasets were applied to bi-dimensional PCA using the Strand NGS PCA tool (Agilent). Each sample score from the covariance matrix was plotted in the 2 eigenvectors PC1 and PC2. For analysis of the hierarchical relationship among the samples, we generated a cluster dendrogram of the gene lists for each sample using an R ‘gplots’ package (http://cran.r-project.org/web/packages/gplots/index.html). To determine the optimum number of k-means clusters, we used the hierarchical cluster datasets of each gene, and further grouped the determined 7 k-means subclusters of co-expressed genes into 2 general patterns. The first subcluster comprised genes that showed highly variable expression in female (n = 5) and male (n = 4) PGCs. The second subcluster comprised genes showing uniform expression in female (n = 2) and male (n = 3) PGCs. We performed GO analysis using the DAVID web tool (http://david.abcc.ncifcrf.gov/) [24] for each gene set of the subclusters.

### Accession number

The RNA-seq and ChIP-seq data from this study have been deposited in the DNA Data Bank of Japan (DDBJ) under the accession number DRA003597 and DRA003803.

## Results

### Summary of gene expression profiling

**Fig 1:**
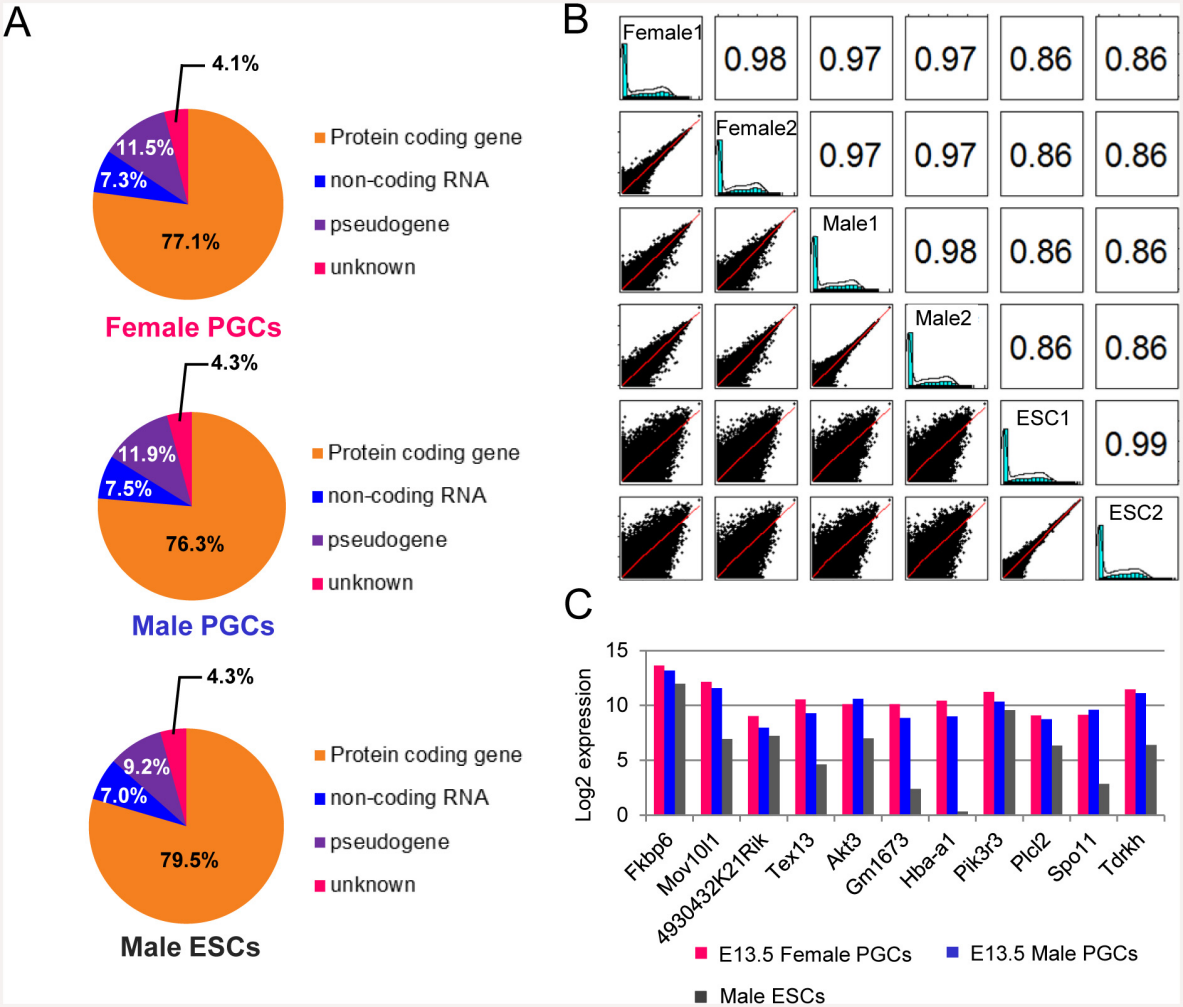
RNA-seq transcriptional profiling of female and male PGCs. **(A)** Pie charts showing the composition and quantity of each reference gene type in the UCSC Genome Browser for each RNA-seq dataset. **(B)** Comparative analysis of reference gene expression levels in each RNA-seq dataset. The left side-scatter plots display the relationship between each gene expression pattern. The right side numbers are the Pearson’s correlation coefficients. **(C)** PGC-specific gene expression patterns (11) in each RNA-seq dataset. Expression levels are shown in log2 values.

The present RNA-seq analysis yielded 27.1–35.6 million unique aligned reads for PGCs and ES cells (Table 1), for which the mapping rates were 70.18%–82.67%, respectively. The Y chromosome was removed from this analysis because the influence of this sex chromosome was excluded. The average number of transcripts detected was slightly higher in male PGCs compared with female PGCs (13,176 vs. 12,611 respectively; duplicate experiments), and the number of transcripts in ES cells was comparable with male PGCs (13222 and 13508, respectively). Assignment to the mouse GRCm38/mm10 reference genome showed that nearly 80% of transcripts encoded proteins, with non-coding genes and pseudogenes each accounting for 10% of genes in all profiles (Fig 1A). The correlation coefficient among all PGC transcriptome profiles was very high (r = 0.97–0.98; Fig 1B). The correlation value between PGCs and ES data sets was somewhat low (r = 0.86), reflecting differences in the respective features of these cells.

**Table 1:** Alignment and quantification statistics in each RNA-seq library sample.

Library	Total reads	Number of reads after trim	Percentage trimmed	Unique alignment reads	Percentage uniquely aligned	Detected all transcripts
E13.5 female PGC lib_1	42729239	42258517	98.9	30889817	73.1	12867
E13.5 female PGC lib_2	42805807	42260667	98.73	30104269	71.2	12354
E13.5 male PGC lib_1	38885293	38523543	99.07	27107722	70.4	13247
E13.5 male PGC lib_2	41244689	40831723	99	28656310	70.2	13104
mESC lib_1	50000000	43082893	86.17	35616704	82.7	13222
mESC lib_2	50000000	43227212	86.45	33783928	78.2	13508

Eleven PGC-specific marker genes [26] were expressed at similar levels in female and male PGCs (Fig 1C), of which 3 genes, *Fkbp6*, *4930432K21Rik* and *Pik3r3*, showed similar expression in ES cells. These findings demonstrate that the transcriptome datasets obtained were of high quality and valid for further analysis.

### Genes specifically expressed in female and male PGCs

**Fig 2:**
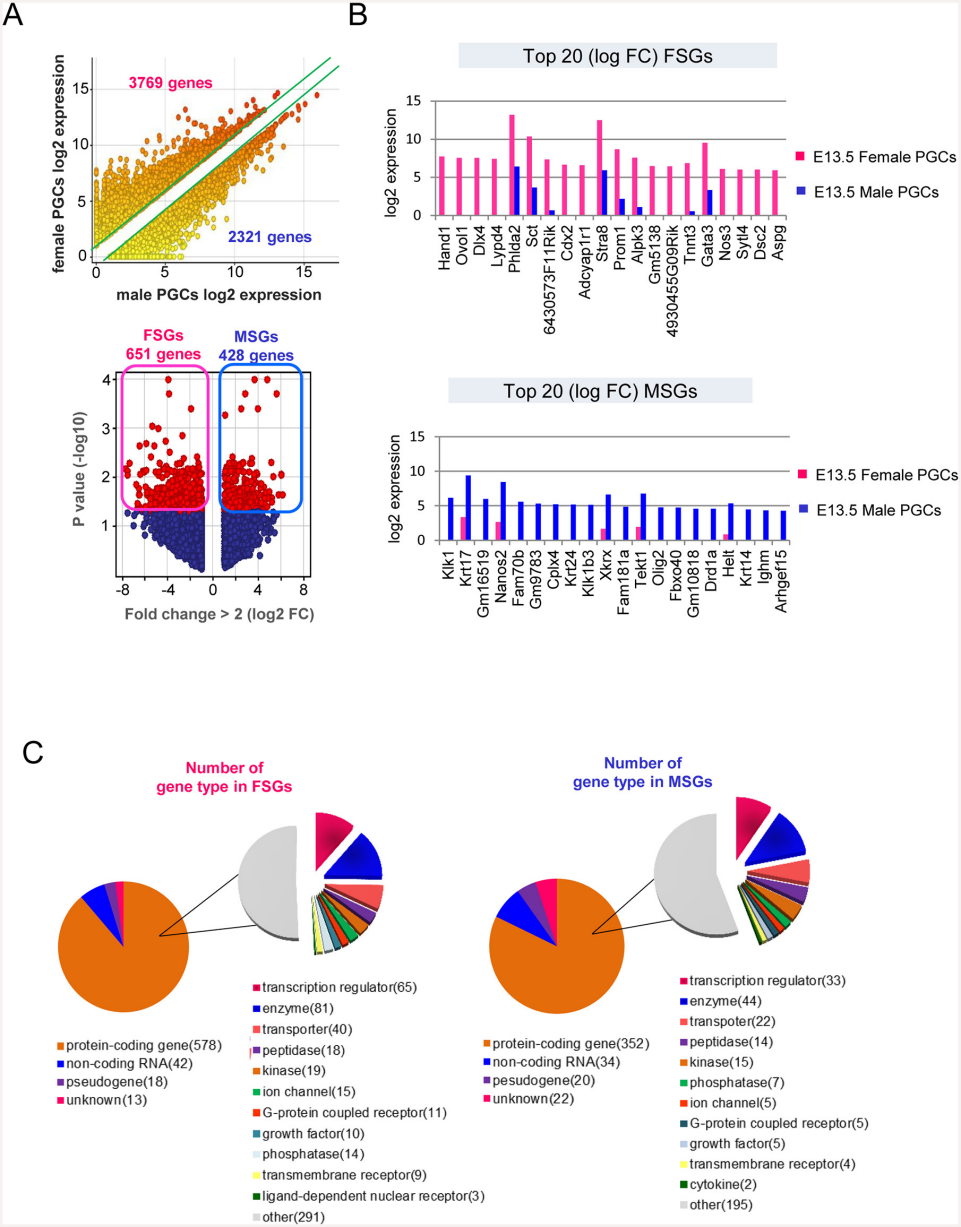
Identification of female and male PGC-specific-expressed genes. **(A)** Top: scatter plot of female and male PGC samples. The green lines indicate a 2-FC in the average expression levels between the 2 samples. Bottom: volcano plot of differentially expressed genes in female and male PGC samples. Red spheres indicate statistically significant genes. FSGs (651) and MSGs (428). **(B)** Top 20 differentially expressed genes in female- and male-specific PGCs. Expression levels are shown in log2 values. The genes are ranked in the order of decreasing log FC values. **(C)** Pie chart showing the composition and quantity of each reference gene type in the UCSC Genome Browser and Ingenuity Pathway analysis tool for FSGs and MSGs.

The sequencing data provided accurate gene expression profiles. Therefore, we conducted an analysis to select genes specifically expressed in female and male PGCs. Genes were screened based on 2 parameters, namely a fold change (FC) of >2 and the statistical significance determined using a moderated *t* test with a Benjamini—Hochberg FDR of <0.05. Based on FC selection criterion (FC > 2), more than 3,700 and 2,300 genes were preferentially expressed in female and male PGCs, respectively (Fig 2A). To eliminate inaccuracies, we selected statistically significant genes (P < 0.05) to identify PGC-expressing FSGs and MSGs. The number of FSGs was 651 (5.2% of the total transcripts) and the number of MSGs was 428 (3.3% of the total transcripts; Fig 2A, S2 Table). The 20 FSGs and MSGs with the greatest statistical significance are shown in Fig 2B. Reference gene types expressed in the FSGs and MSGs were similar, with 88.8% and 82.2% of the protein-coding genes enriched with transcriptional regulators, enzymes, and transporters, respectively (Fig 2C).

### Transcription factor expression

A large number of transcription factors (TFs) was expressed in female and male PGCs at E13.5 (female: 1052, male: 993). FSGs and MSGs contained 65 and 33 TFs, respectively, which were selectively activated in either female or male PGCs (S2 Fig). Many of the TFs were classified as members of the homeobox family (16 in FSGs and 7 in MSGs); 8 FSGs and 3 MSGs belong to the C2-H2 zinc-finger family. Based on an *in silico* investigation (Mouse Genome Informatics), no clear expression of these TFs was detected in germ layers of E13.5 mouse embryos. The finding that these gene expression levels were enhanced in PGCs at this time point suggested that the TFs identified are involved in sex-specific differentiation. Interestingly, we identified 5 cofactors in FSGs (*Tgfb1i1*, *Cited1 Grip1*, *Nfkbia*, and *Lmo4*) and 4 in MSGs (*Epas1*, *Optn*, *Actn2*, and *Btg1*), suggesting that these cofactors regulate the activity of their respective target TFs. To date, little is known regarding the functions of FSGs and MSGs in PGCs. Thus, it is difficult to explain the exact roles of these TFs in PGC development.

### Genes regulating DNA and histone methylation

Expression of epigenetic regulator genes was evaluated. *Dnmt3a* was clearly expressed in both female and male PGCs, and ES cells. However, *Dnmt3l* was not expressed in female PGCs (S2 Fig). These observations were coincident with the fact that *de novo* DNA methylation was absent in female PGCs during foetal stage. Genes regulating DNA demethylation, such as *Prdm14*, *Tet1*, *and Tet2*, were expressed at similar levels in female and male PGCs, as well as in ES cells. However, *Tet3* expression was low, and expression of the activation-induced cytidine deaminase gene (*Aicda*) was not detected. The numbers of genes regulating H3K4me3 and H3K27me3 methylation was similar in female and male PGCs, as well as in ES cells. These findings support the hypothesis that histone modifications serve as major regulators of sex-specific gene expression in PGCs.

### GO and network analysis

**Fig 3:**
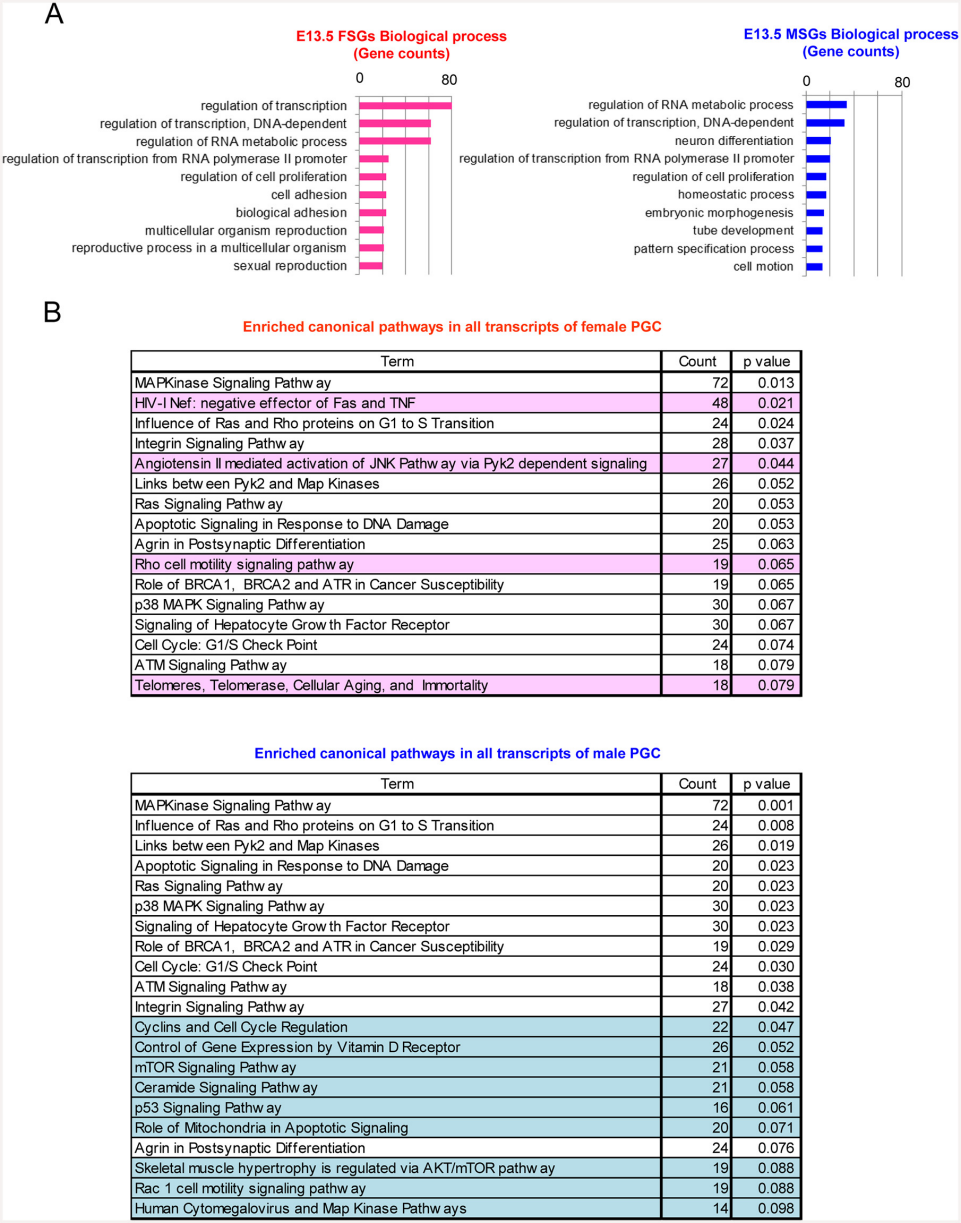
Biological significance of female and male-specific PGC-expressed genes. **(A)** GO enrichment analysis of FSGs and MSGs. The most highly enriched biological processes based on their respective gene counts are shown (Fisher’s exact test: cut-off < 0.1). **(B)** Pathway analysis of all transcript lists in female and male PGCs. The lists indicate enriched pathways observed among female and male transcripts, as determined using DAVID (Fisher’s exact test: cut-off <0.1). Sex-specific pathways are highlighted in pink and blue, which denote female- and male-specific, respectively.

To gain a better understanding of the biological functions of FSGs and MSGs, we performed gene ontology and network analyses (Fig 3A and 3B), using DAVID. GO analysis revealed significant functional differences between FSGs and MSGs (Fig 3A). Among the FSGs (n = 651), 80 (12%), 62 (10%), and 62 (10%) were annotated to ‘regulation of transcription’, ‘regulation of transcription, DNA dependent’, and ‘regulation of RNA metabolic process’, respectively. A similarly strong enrichment for specific GO terms was not observed with the MSGs. Of the MSGs (n = 428), 35 (8%), 32 (7%) and 22 (5%) were annotated to ‘regulation of RNA metabolic process’, ‘regulation of transcription, DNA-dependent’, and ‘neuron differentiation’, respectively.

To gain further insights into the differences between the biological functions of female and male PGCs, we performed a gene-network analysis using the BioCarta program. Using all transcripts detected in each of the duplicate datasets, 16 and 21 networks were formed in female and male PGCs, respectively (Fig 3B, S3 Fig). Of these, 4 and 9 sex-specific networks were activated in female and male PGCs, respectively. Interestingly, some of the identified networks are involved in apoptosis and cell death. For female PGCs, the following networks were identified: ‘HivnefPathway: negative effector of Fas and TNF’ and ‘telPathway: Telomeres, Telomerase, Cellular Aging, and Immortality’ (S3D Fig). For male PGCs, the following networks were identified: ‘ceramidePathway: Ceramide Signaling Pathway‘, ‘mitochondriaPathway: Role of Mitochondria in Apoptotic Signaling’, and ‘p53Pathway: p53 Signaling Pathway’ (S3E Fig). In the ‘HivnefPathway‘ (S3D Fig), among 48 genes, *Casp6*, *Traf1*, and *Nfkbia*, which are critical constituent genes of the TNF-TNF receptor pathway, were specifically expressed in female PGCs. Importantly, *Casp6* is an apoptosis effector [27]. In male PGCs, 3 pathways were formed, in which 16–21 genes were enriched (Fig 3B). One of the formed pathways was ‘ceramidePathway’ ceramide is a sphingosine-based lipid-signalling molecule located in the cell membrane, which functions as a pro-apoptotic regulator [28]. Mitochondria participate in apoptotic signalling pathways through the release of mitochondrial proteins into the cytoplasm [29, 30]. Cells with damaged DNA are eliminated by p53-dependent processes (S3C Fig) [31, 32]. Therefore, some DNA damage could have been present in the male PGCs. These results indicate that the PGC-attrition program could have been activated in both female and male PGCs at E13.5 via different pathways.

A strong enrichment was observed in MAP kinase-related and cell cycle-related pathways in female and male PGCs (Fig 3B, S3B Fig). These networks are potentially involved in cell cycle regulation. Our results suggest that neither female nor male PGCs entered cell cycle arrest. Interestingly, the ‘RasPathway’, which is involved in cell survival, was also formed in female and male PGCs (S3A Fig). The ‘cellcyclePathway’, which formed in male PGCs, showed that genes involved in all cell cycle stages were activated at E13.5, suggesting that these cells were still at the mitotic stage (Fig 3B). These enriched GO terms and networks provide a new perspective on the distinctive features of female and male PGCs. The results strongly support the notion that the sex-specific fate of PGCs is determined by E13.5.

### Epigenetic regulation for MSGs and FSGs

**Fig 4:**
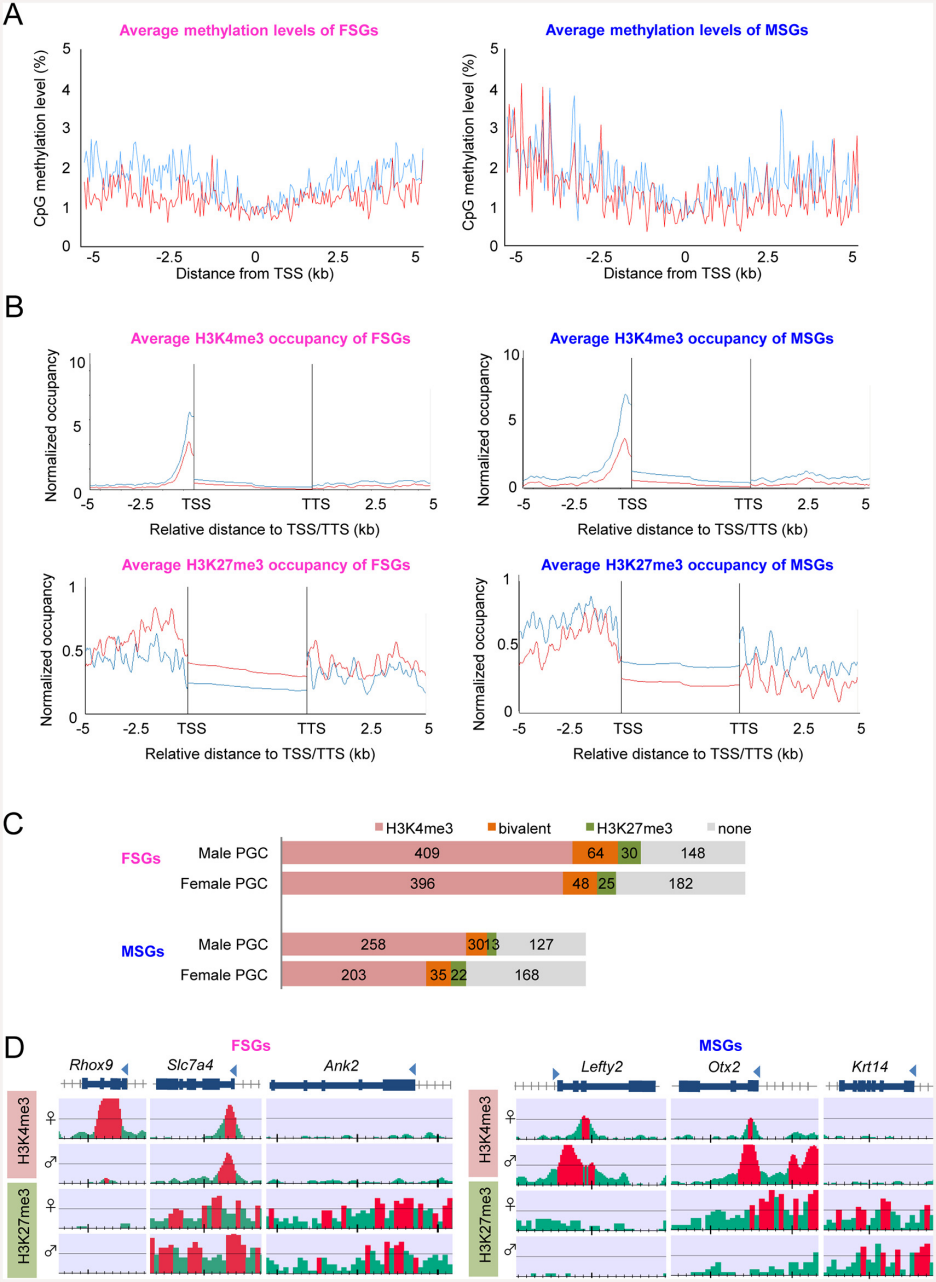
DNA methylation and histone modifications in female- and male-specific PGC-expressed genes. **(A)** Average methylation levels of TSS and the shore values (±5 kb of the TSS) in female- and male-specific PGC-expressed genes. Our previous methylation data of PGCs were obtained from the DDBJ database [12]. **(B)** Occupancy of H3K4me3- and H3K27me3-bound TSS and TTS shore regions (±5 kb of the TSS and TTS) in female- and male-specific PGC-expressed genes. **(C)** Proportion of genes showing histone-modification patterns. The number of genes classified into each histone-modification pattern is shown. **(D)** Three typical histone-modification patterns. The red regions indicate ChIP/input enrichment-peak regions that were calculated using the DROMPA peak-calling program.

A question is whether epigenetic mechanisms regulate sex-specific gene expression in PGCs. To address this, we evaluated the DNA methylation status of the FSG and MSG promoter regions using our previous single-resolution methylome datasets obtained using the post-bisulphite adapter tagging (PBAT) method [12]. We confirmed that these regions were entirely hypomethylated (<3%) in both female and male PGCs (Fig 4A). These findings showed that DNA methylation does not regulate FSGs and MSGs. Next, to investigate potential epigenetic regulatory mechanisms, we performed a ChIP-seq analysis for H3K4me3 (an active marker) and H3K27me3 (a repression marker), as shown in Fig 4B. The promoter regions of FSGs (n = 651) and MSGs (n = 428) were clearly marked with H3K4me3 in both female and male PGCs. H3K27me3 was enriched on the promoter and shore regions in female and male PGCs, with a slightly higher accumulation observed in FSGs of male PGCs, and in MSGs of female PGCs.

The numbers of FSGs and MSGs that showed a peak for H3K4me3 and/or H3K27me3 were analysed using the MAX peak-calling program (Fig 4C). The FSGs patterns related to histone modifications were similar between the female and male PGCs: Over 60% of the FSGs were marked with H3K4me3, and a bivalent status displaying both active and negative histone markers was detected in 7.4 and 9.8% of the genes in female and male PGCs, respectively. Similar modification patters were also observed in MSGs. Interestingly, 22–39% of genes were neither marked with H3K4me3 nor H3K27me3. Together with the observation that no differences in histone modification occurred between female and male PGCs in some genes, our results suggested that other histone modifications are responsible for sex-specific gene expression in the PGCs.

Three representative cases are shown in Fig 4D. The promoter regions of *Rhox9* (an FSG) and *Lefty2* (an MSG) were heavily marked with H3K4me3 in female and male PGCs, respectively. These facts suggested that H3K4me3 is a major regulator in the sex specific expression of these genes. Bivalent modifications of the FSG *Fgfr3* and the MSG *Otx2* of MSGs were observed, and H3K27me3 appeared to function as a repressive marker of *Otx2* in female PGCs. Furthermore, the FSG *Dnajb8* and the MSG *Krtl14* were marked with H3K27me3 within the repressed sex of PGCs. These findings indicated that histone modifications are potential regulators of FSG and MSG expression in E13.5 PGCs.

### Imprinted gene expression

Expression of 7 maternally and 10 paternally expressed imprinted genes was detected in both female and male PGCs (S4 Fig). Interestingly, expression of *Igf2* and *Dlk1* was significantly higher in female PGCs compared with male PGCs. Most of the imprinted genes were expressed at levels similar to those observed in ES cells, with the exception of *Igf2*, *Meg3*, *Dlk1*, *Peg10*, *Sgce*, and *Cdkn1c*, which were either expressed 3-fold higher or 2-fold lower in PGCs (S4A Fig). To identify the associated expression alleles, we used PGCs derived from B6 × DBA and DBA × B6 mouse foetuses for single nucleotide polymorphism (SNP) detection. The sequencing results showed that *Snrpn*, *Ube3*, and *Dlk1* displayed bi-allelic expression in female PGCs. However, these genes showed imprinted expression in male PGCs (S4B Fig).

### Single cell transcriptome

**Fig 5:**
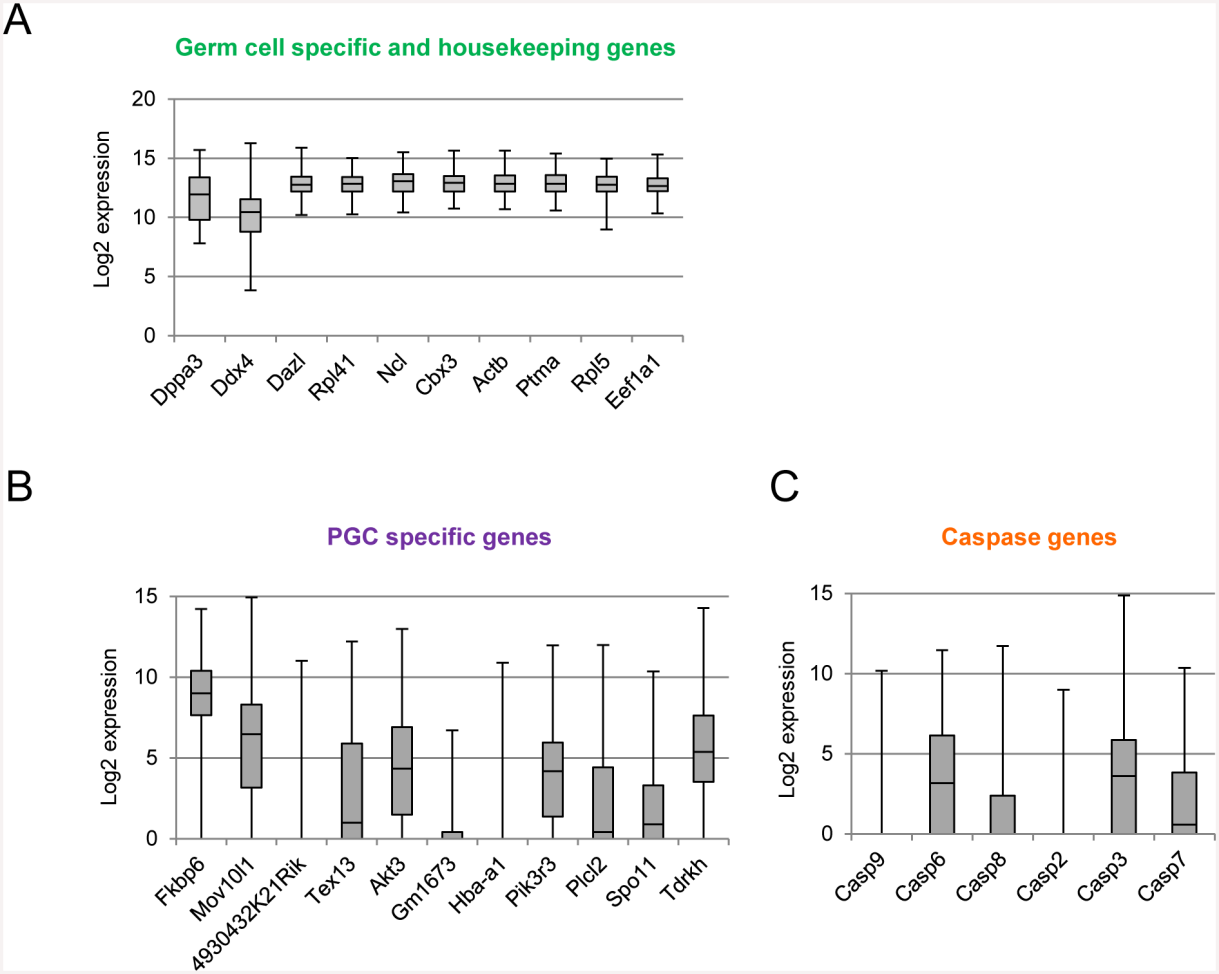
Expression patterns of representative genes in individual PGCs. **(A)** Uniform expression patterns of housekeeping and germ cell-specific genes among individual PGCs. **(B, C)** Heterogeneous expression pattern of PGC-specific and apoptosis-related genes. Expression levels are shown in log2 values. Gene symbols were ranked in the order of higher log (FC) values. Box plots show the central 75% (grey boxes), median (black lines), and range (whiskers).

**Fig 6:**
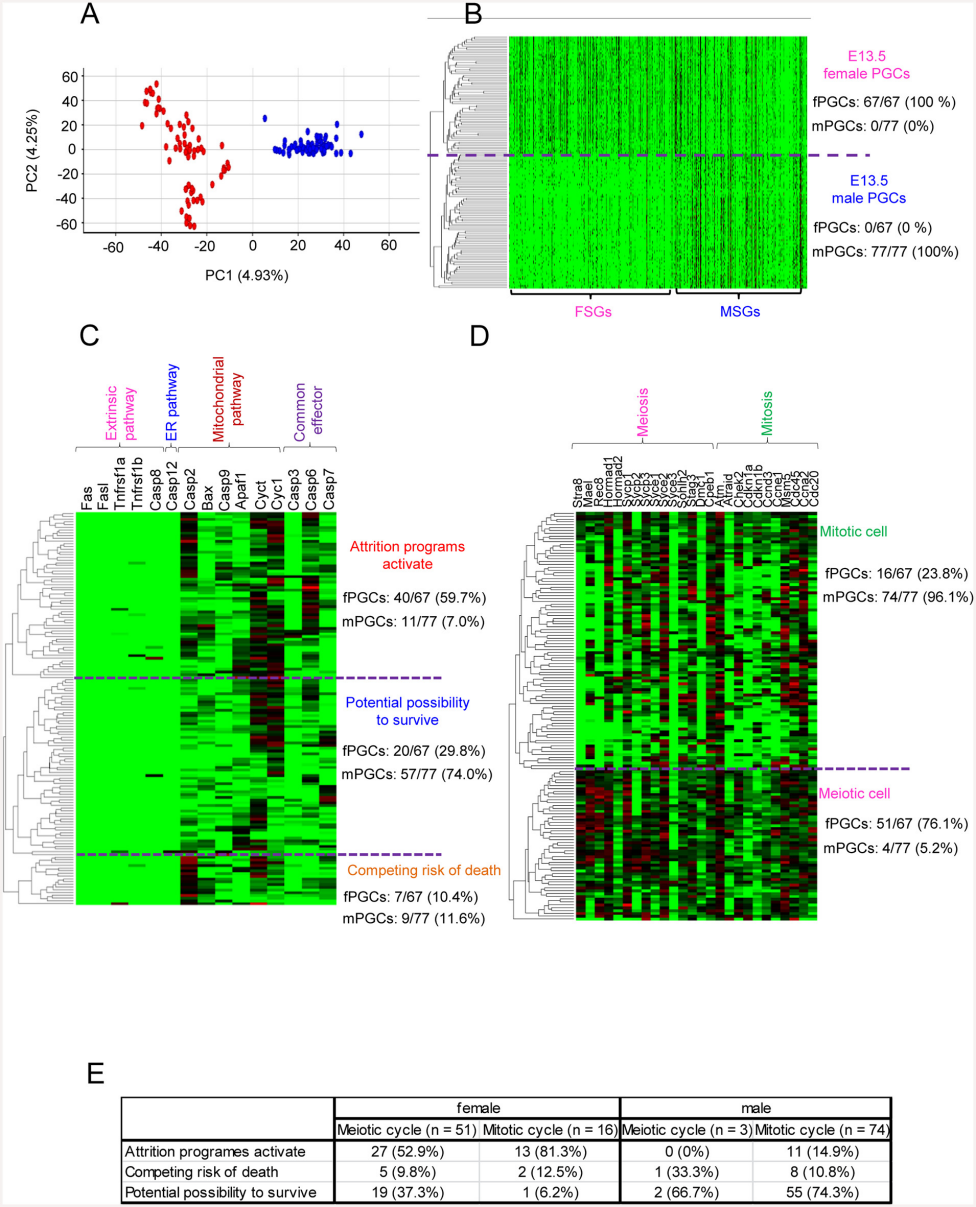
Single-cell transcriptome analysis of E13.5 female and male PGCs. **(A)** Bi-dimensional **PCA** of E13.5 female and male single-PGC gene expression patterns. The first principal component (PC1) captures 4.93% of the gene expression variability and the second principal component (PC2) captures 4.25%. The red and blue spheres represent single female (n = 67) and male (n = 77) PGCs, respectively. **(B)** Hierarchical clustering analysis of transcriptome data sets (FSG: 651, MSG: 428) of female and male single PGCs. **(C)** Heat map of the relative expression of apoptotic process-related genes (n = 15). **(D)** Heat map of the relative expression of meiotic and mitotic cell cycle-related genes (n = 26). **(E)** Relation between apoptosis and meiotic and mitotic cell cycle in female PGCs. The numbers indicate the number of PGCs.

The present transcriptome data demonstrated clear differences in the sex specification of PGCs. However, the heterogeneity of PGCs remains unknown. To address this issue, we conducted a single-cell transcriptome analysis, in which datasets from 67 female and 77 male PGCs were obtained. The mean number of transcripts detected was 6593 (range: 3787–8763; S3 Table). Expression of germ cell-specific genes and housekeeping genes was normal, except in a few cases (Fig 5A). Clustering analysis and PCA demonstrated that the transcriptome datasets were perfectly separated into 2 clusters of female and male PGCs (Fig 6A and 6B). Transcriptome datasets of female PGCs varied considerably in the second principle component. The correlation coefficient values ranged from 0.67 to 0.96 in female PGCs and from 0.53 to 0.81 in male PGCs. (S5 Fig). To investigate the type of genes that contributed to the transcriptional heterogeneity in female and male PGCs, we performed k-means clustering analysis for each gene expression level, as well as GO analysis with genes in the respective subclusters (S6 Fig). Interestingly, different GO terms were enriched in subclusters of the female and male PGCs. For instance, in female PGCs, cell cycle- and cell death-related GO terms were enriched in subclusters 3 (highly variable gene set) and subclusters 4 (uniform gene set), respectively. These findings clearly demonstrated the heterogeneity of the female and male PGC populations.

These results were supported by the gene-network analysis results (Fig 3B, S3 Fig). Interestingly, some genes (*Fas*, *FasL*, *Tnfrsf1a*, *Tnfrsf1b*, *Casp8*, and *Casp12*) involved in the extrinsic and endoplasmic reticulum-based apoptosis pathways were not enhanced in both female and male PGCs. However, the mitochondrial pathway and common effector caspases were activated in 59.7% of female PGCs, while the mitochondrial pathway was activated in only 7% of male PGCs (Fig 6C). These findings suggested that these cells had initiated apoptosis. Furthermore, genes related to meiosis (*Stra8*, *Mael*, *Rec8*, *Sycp1*, and *Sycp3*) were enhanced in 76.1% of female PGCs, but only 5.2% of male PGCs (Fig 6D). Of meiotic and mitotic female PGCs, 62.7 and 93.8% expressed apoptosis-related genes, respectively (Fig 6E). In contrast, the majority of male PGCs (96.1%) were likely at the mitotic stage, as genes involving the cell cycle were activated. Interestingly, 10.8% of male PGCs expressed G0 arrest-related genes, including *Atm*, *Chek2*, *Cdkn1a*, and *Cdkn1b*. These findings revealed the heterogeneity of E13.5 female and male PGCs. Thus, the single-cell transcriptome datasets showed a process of continuous fate determination.

## Discussion

We obtained transcriptome data by performing deep RNA-seq analysis and generated female- and male-specific PGC gene expression sets. Our transcriptome data demonstrated that complex gene networks become independently established in female and male PGCs by E13.5. These data provide new insights into the molecular signatures associated with the assignment of sex-specific fates in PGCs.

To date, only a few studies have focused on PGC sex specification. In the genital ridge, granulosa cells in females and Sertoli and Leydig cells in males become differentiated in association with transient *Sry* expression at E11.0–12.0 [6, 7]. Enhanced expression of genes related to meiotic division and Nodal signalling is detected in female and male PGCs, respectively [33–36]. Indeed, PGCs are known to be bipotential until E11.5, meaning they are able to differentiate into germ cells of either sex. When bipotential PGCs are transplanted into recipient gonads of the opposite sex, XX and XY PGCs adapt to the sex-reversal environment and differentiate into spermatozoa and oocytes, respectively [7]. Jameson *et al*. conducted a PGC study investigating gene expression profiles in mice using microarrays [11]. The authors screened for primed genes, which were specifically deficient or enriched in each lineage, along with sex specification in PGCs, supporting cells, interstitial/stromal cells, and endothelial cells. To gain further insight into the sex specification of E13.5 PGCs, we compared FSGs and MSGs identified by our RNA-seq data with the primed genes reported by Jameson et al. Importantly, of the sex-specific genes reported in the previous study, only a limited number were found in FSGs and MSGs in the present study (27 and 42 genes, respectively). Thus, the present findings provide novel insights into the sex-specific features of PGCs. Therefore, we performed further analyses to understand the biological functions of the FSGs and MSGs in PGC sex specification.

Interestingly, many TFs were detected in FSGs (65 genes) and MSGs (33 genes). Of these, several TFs belonged to the homeobox family containing helix-turn-helix motifs. This family of TFs directly binds the regulatory elements of related target genes and promotes the transcription of genes associated with the regulation of cell differentiation and organogenesis. Therefore, homeobox-family TFs in FSGs and MSGs could directly act upon their target genes and play important roles in sex-specific fate determination in PGCs. Interestingly, caudal type homeobox 2 (*Cdx2*) and heart and neural crest derivatives expressed transcript 1 (*Hand1*), which are involved in the differentiation of trophoblastic cell lines [37, 38], were exclusively expressed in female PGCs. *Cdx2* expression is inhibited by *Oct3/4* in the inner cell mass at the blastocyst stage and in ES cells [39, 40]. The roles of *Cdx2* and *Hand1* in PGCs are presently unknown. However, these TFs could be involved in PGC sex specification via *de novo* regulation networks. Many TFs involved in the differentiation and proliferation of neural cells were highly expressed in male PGCs. The Notch signal-response pathway, which regulates the differentiation and conservation of neural stem cells [41, 42], is functional in Sertoli cells differentiated in the genital ridge, following *Sry* expression [43]. These observations suggest that male PGCs respond to an inhibitory signal to differentiate from Sertoli cells. We also identified various TF cofactors in FSGs and MSGs. *Tgfb1i1*, *Cited1*, *Grip1*, *Nfkbia*, and *Lmo4* were expressed in female PGCs, whereas *Epas1*, *Optn*, *Actn2*, and *Btg1* were expressed in male PGCs. Interestingly, the TFs interacting with these cofactors were highly expressed in both female and male PGCs. This suggests that these cofactors regulate the expression of FSGs and MSGs. Genome-wide demethylation in PGCs may provide an environment that causes leaky expression of some genes; therefore, protein expression in PGCs merits additional study.

Moreover, our RNA-seq data showed that expression of *Aicda* was not expressed in both female and male PGCs. This result suggests that DNA demethylation occurring via deamination of 5mC was not functional, although it has been reported that *Aicda*-deficient PGCs showed greater methylation than wild type PGCs, particularly in introns and transposons at E13.5 [44]. A model supported by this recent study is that 5mC of PGCs is not converted to 5-formylcytosine (5fC) and 5-carboxylcytosine (5caC) [45]. Therefore, demethylation in PGCs may have occurred via DNA replication accompanied by cell division.

Our GO and gene-network analyses provided additional evidence of biological differences occurring between female and male PGCs that were activated at E13.5. The ‘HivnefPathway’ facilitates the escape of HIV-infected cells from apoptosis, whereby the viral NEF protein plays a key anti-apoptotic role by repressing *Ask* expression (S4 Fig) [46]. The interaction between NEF and ASK1 prevents phosphorylation of downstream MAP and JNK kinases involved in apoptotic signalling. However, the present transcriptome data did not reveal the expression of genes related to the extrinsic pathway of apoptosis, such as *Tnf*, *Faslg*, and *nef*, in female PGCs. These transcriptome data suggested that apoptosis of female PGCs was induced by an intrinsic apoptosis pathway, such as the mitochondrial pathway. This possibility is supported by our single-cell transcriptome analysis results, which showed that the PGCs expressed genes involved in the intrinsic pathway. Pepling et al. reported that few female PGCs at E13.5 were positive for cPARP, which is a maker of late-stage apoptosis [47]. However, the present results suggested that apoptosis was already initiated by the intrinsic pathways in a large proportion of the female PGCs.

Unexpectedly, 3 pathways involving apoptosis were observed in male PGCs. Of these, the ‘ceramidePathway’ and ‘mitochondriaPathway’ were of interest, as they play a significant role in apoptosis. Ceramide is a sphingosine-based membrane-associated lipid signalling molecule that functions as a pro-apoptotic regulator [28]. Mitochondria participate in apoptotic signalling pathways through the release of mitochondrial proteins into the cytoplasm [29, 30]. These 2 pathways could interact with each other, as ceramide activates mitochondrial permeability, leading to cytochrome c release and apoptosis induction. Although little is known regarding apoptosis in male PGCs, findings from an electron microscopy study showed typical apoptosis features, such as nuclear fragmentation, were observed in 50% of the cells. However, the total number of apoptotic cell has not yet been shown in mice [48].

PGCs quickly proliferate while migrating into the genital ridge, with their number reaching a maximum of approximately 12,000 at E13.5 [49]. Once apoptosis is initiated, the number of PGCs decreases in females and males [50]. The number of oocytes and gonocytes are thought to decrease during the embryonic period [47, 48, 51]. Although little information is available to explain how this decrease in cell number occurs, the elimination process could be associated with the removal of potentially abnormal germ cells. Previous data indicated that the apoptosis pathway was not activated in female and male PGCs at E13.5. Exactly how the PGC/oocyte-attrition process is initiated is poorly understood. Our network analysis of the RNA-seq datasets from pooled PGCs indicated that latent apoptosis pathways were already activated in female and male PGCs by E13.5. In parallel, pathways related to survival were also activated in both female and male PGCs, including the ‘RasPathway’ and ‘MAPKinasePatheway’. Several cell cycle-related pathways were enriched in both female and male PGCs. The ‘cellcyclePathway’ formed in male PGCs, showing that genes involved in all cell cycle stages (G1, S, G2 and M) were activated at E13.5, suggesting these cells were still at the mitotic stage. This possibility is supported by the results of our single-cell transcriptome analysis: male PGCs were at mitotic stage except for a few cells, while the majority of female PGCs were already at the meiotic stage. These results suggested that the PGCs in our study comprised a heterogeneous cell population, consisting of both apoptotic and non-apoptotic cells.

Recent data have indicated the importance of obtaining global molecular information at a single-cell resolution, which can reveal the heterogeneity of cell populations and enable identification of cancer precursor cells [52]. To gain further insight into PGC features, we conducted a single-cell transcriptome analysis. Together with the gene-network analysis, our results revealed several unique features of PGCs at E13.5. Firstly, the PGCs existed as a heterogeneous cell population. Secondly, a large number of female PGCs were potentially undergoing apoptosis. Thirdly, the majority of female PGCs shifted to the early phase of meiosis. The number of PGCs at E13.5 has been reported to be 24,000 [53]. However, a more reliable analysis recently showed that this number was 12,000 and that, as a consequence of cell attrition, this number was reduced by 1/3 at birth [47]. Interestingly, our single-cell transcriptome analysis showed that almost 60% of female PGCs expressed apoptosis-related genes. Therefore, these PGCs could be eliminated from the ovaries around the time of birth. A lower proportion of PGCs (14%) was undergoing the apoptosis process in male PGCs. Histological results indicated that 50% of male PGCs exhibit apoptotic signals at E17.5 [48]. These results showed that each PGC was assigned to a different fate at this stage. As a consequence, PGCs undergo either differentiation into viable germ cells or are subjected to cell attrition during foetal development.

Previously, we conducted single-resolution DNA methylome analyses using the PBAT method to determine whether demethylation influences PGC development [12]. The results revealed that PGCs were uniformly hypomethylated at E13.5, except for at the retrotransposon regions. Next, we determined the hypomethylation status of TSS and the shore regions in FSGs and MSGs. We found that DNA methylation did not regulate the expression of FSGs and MSGs, suggesting that histone modifications were responsible instead. The results of several recent studies using ChIP-seq analysis have suggested that histone modifications regulate gene expression in mouse PGCs [19, 20]. H3K4me3 and H3K27ac, which are active markers, significantly accumulated at the promoter regions of *Zic3* and *Dazl*. Interestingly, repressed genes, such as *Dlx* and *Hoxa*, showed a bivalent status marked by both active H3K4me3 and negative H3K27me3. This bivalent status was also observed in some genes expressed at low levels. The pattern of H3K4me3 and H3K27me3 was also detected in our ChIP-seq datasets. Further ChIP-seq analyses for H3K9me2, H3K9me3, H3K27ac, and other histones would provide further insights into the epigenetic regulation of FSG and MSG expression levels.

In conclusion, data presented in this study demonstrated that PGC sex specification is associated with complex gene networks. In PGCs, these networks play a critical role in the tightly regulated process of sex specification to generate vital germ cells and minimize the risk of developmental embryonic failure.

## Supporting Information

S1 Fig PGC collection and library construction for next-generation sequencing. **(A)** Schematic representation of the construction of RNA-seq and ChIP-seq libraries for analysis of E13.5 female and male PGCs. **(B)** Representative images of PGCs collected by cell sorting and stained with anti-SSEA1 antibodies conjugated with PE (red).
(TIF)

S2 Fig Expression of regulator genes involved in transcription and epigenetic modifications. Expression levels of 98 transcription regulator genes and 20 epigenetic modification-related genes found in female and male PGCs, ES cells, oocytes, and spermatozoa. The intensities of the blue and red colour gradients indicate genes with low and high expression, respectively. RNA-seq data of oocytes and spermatozoa were obtained from our previous datasets.
(TIF)

S3 Fig Representative enriched BioCarta pathway of all transcripts in female and male E13.5 PGCs. Charting pathways of the BioCarta database from female and male E13.5 PGC all-transcript lists. The red stars indicate genes that found in the list of all female or male transcripts. **(A–C)** Representative common pathways in both female and male PGCs: **(A)** Ras signalling, **(B)** MAPK signalling and **(C)** apoptotic signalling in response to DNA damage. **(D)** Female-specific pathway, HIV-1/Nef Pathway. **(E)** Male-specific pathway, ceramide signalling pathway.
(TIF)

S4 Fig Imprinted gene expression in both E13.5 PGCs and mouse ESCs. **(A)** Representative imprinted gene expression patterns from each RNA-seq dataset. Expression levels are described in log2 values. **(B)** Allele-specific RT-PCR sequencing analysis of 4 imprinted loci was performed using BDF1 and DBF1 PGCs at E13.5. SNPs are highlighted in red.
(TIF)

S5 Fig Heat maps with Pearson’s correlation coefficients among transcriptome datasets of female and male PGCs. The intensities of the colour gradients indicate the correlation coefficient values between 2 samples.
(TIF)

S6 Fig Biological significance of highly variable genes and uniform genes in female and male single PGCs. Seven distinct patterns were classified, and the number of genes included in each subcluster is indicated. Expression levels are shown in log2 values, and blue lines indicate the cluster centroid for each subcluster. The most enriched biological processes based on their respective p values are shown (Fisher’s exact test: cut-off < 0.1). **(A)** female PGCs, **(B)** male PGCs.
(TIF)

S1 Table Gene transcript profiling for E13.5 femle and male PGCs, and ESCs by RNA-seq. (XLSX)

S2 Table Lists of respectively sex-specific expressed genes. The genes which overlapped with Jemeson`s report are highlighted in red.
(XLSX)

S3 Table Alignment and quantification statistics in each single cell RNA-seq library sample. (XLSX)

S4 Table Primer sequences and PCR conditions for each imprinted gene. (TIF)
