# Supplementary figures and images for: Sex Specification and Heterogeneity of Primordial Germ Cells in Mice
Source: PLoS One. 2015 Dec 23;10(12):e0144836. doi: 10.1371/journal.pone.0144836 (PMC4689518; doi:10.1371/journal.pone.0144836)

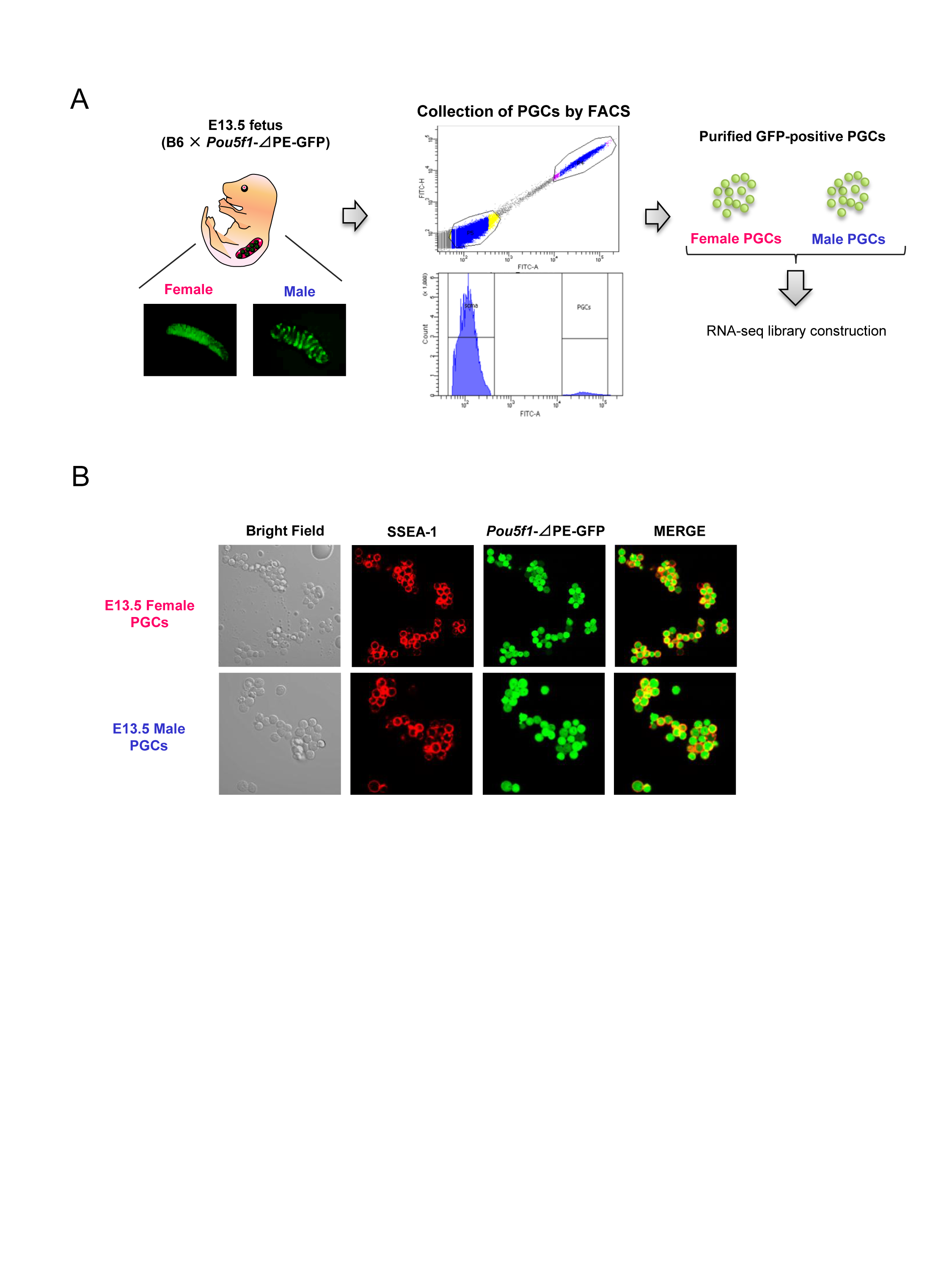

Supplement: S1 Fig — (A) Schematic representation of the construction of RNA-seq and ChIP-seq libraries for analysis of E13.5 female and male PGCs. (B) Representative images of PGCs collected by cell sorting and stained with anti-SSEA1 antibodies conjugated with PE (red). (TIF) [file pone.0144836.s001.tif]

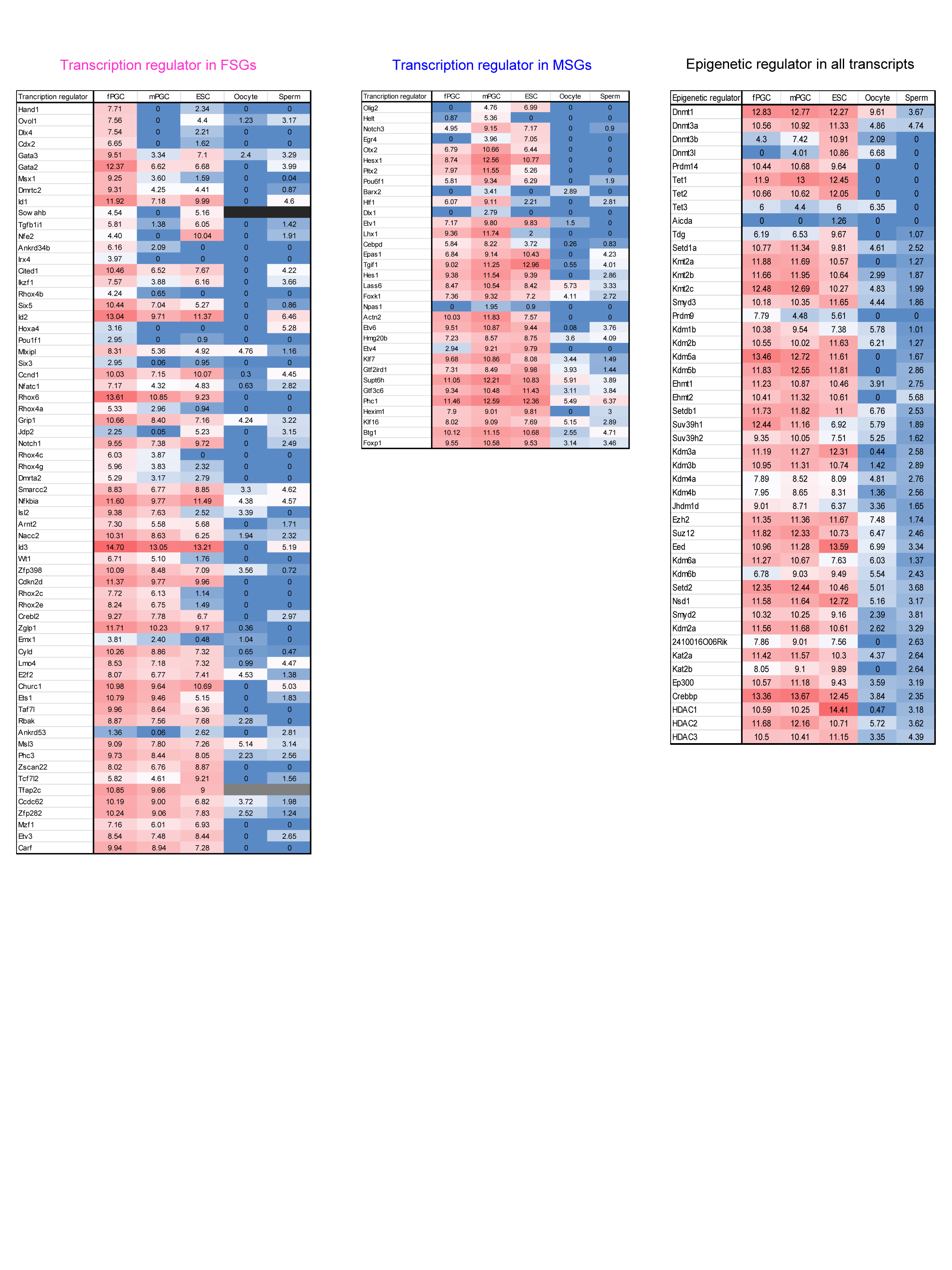

Supplement: S2 Fig — Expression levels of 98 transcription regulator genes and 20 epigenetic modification-related genes found in female and male PGCs, ES cells, oocytes, and spermatozoa. The intensities of the blue and red colour gradients indicate genes with low and high expression, respectively. RNA-seq data of oocytes and spermatozoa were obtained from our previous datasets. (TIF) [file pone.0144836.s002.tif]

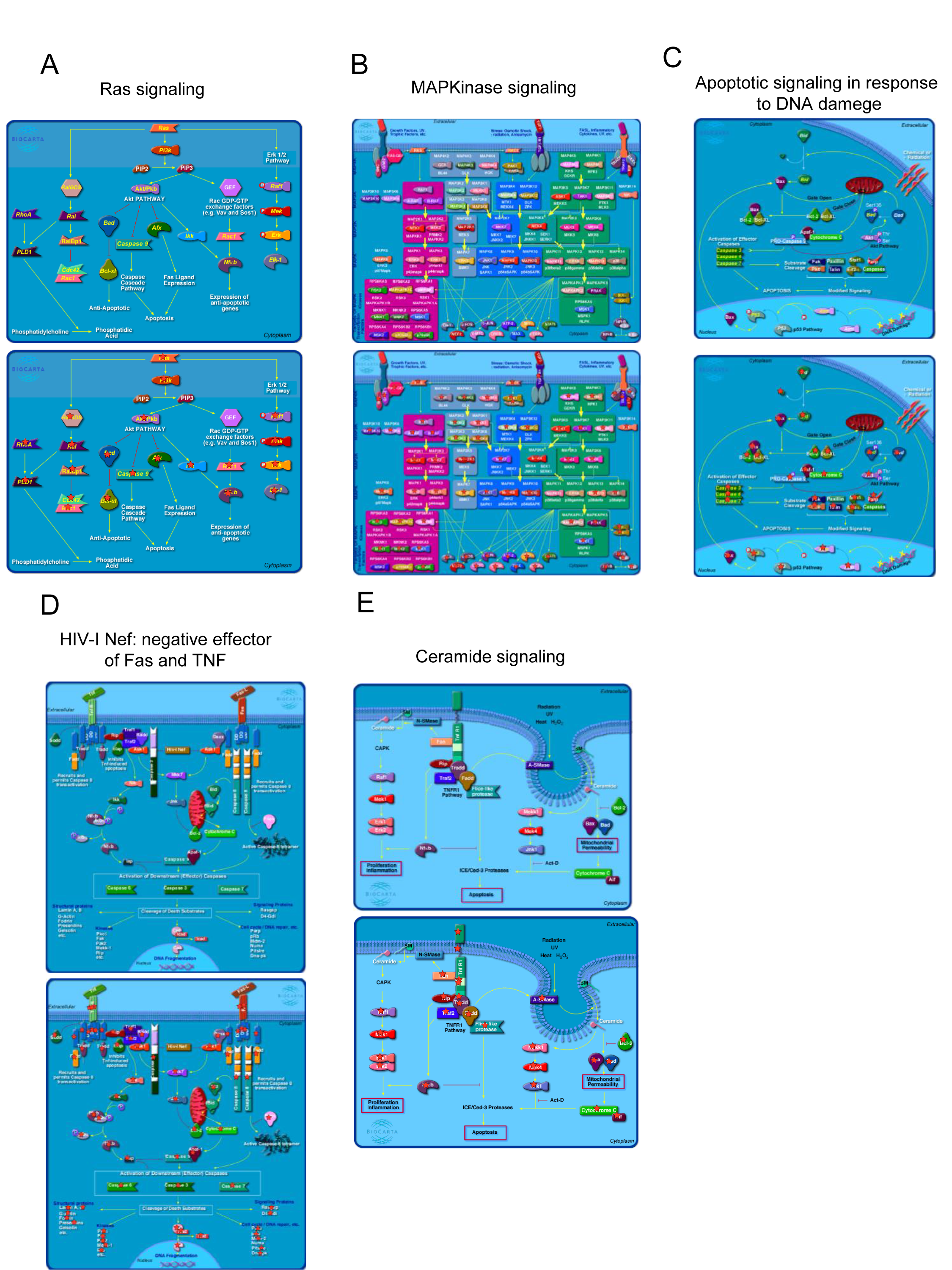

Supplement: S3 Fig — Charting pathways of the BioCarta database from female and male E13.5 PGC all-transcript lists. The red stars indicate genes that found in the list of all female or male transcripts. (A–C) Representative common pathways in both female and male PGCs: (A) Ras signalling, (B) MAPK signalling and (C) apoptotic signalling in response to DNA damage. (D) Female-specific pathway, HIV-1/Nef Pathway. (E) Male-specific pathway, ceramide signalling pathway. (TIF) [file pone.0144836.s003.tif]

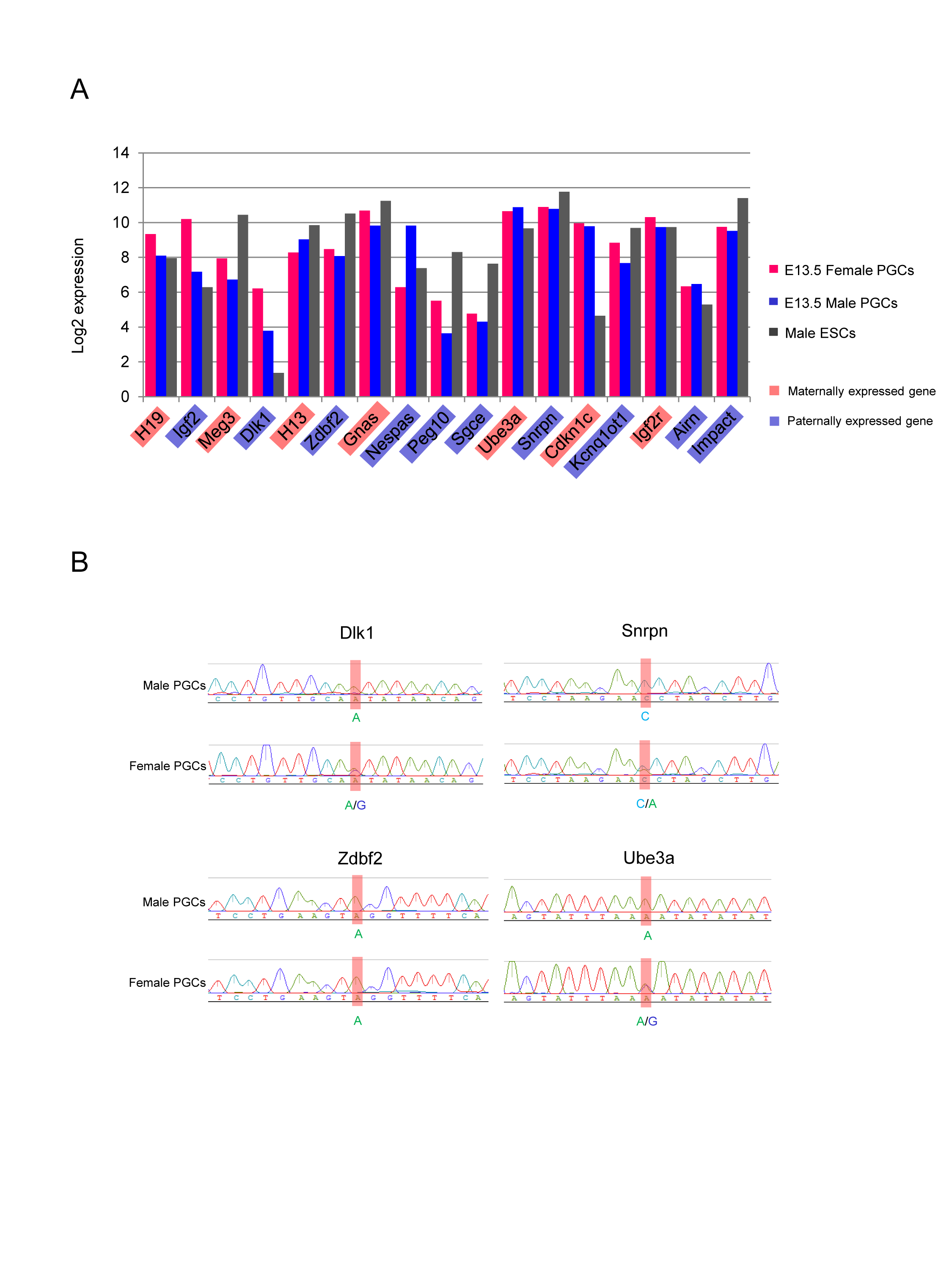

Supplement: S4 Fig — (A) Representative imprinted gene expression patterns from each RNA-seq dataset. Expression levels are described in log2 values. (B) Allele-specific RT-PCR sequencing analysis of 4 imprinted loci was performed using BDF1 and DBF1 PGCs at E13.5. SNPs are highlighted in red. (TIF) [file pone.0144836.s004.tif]

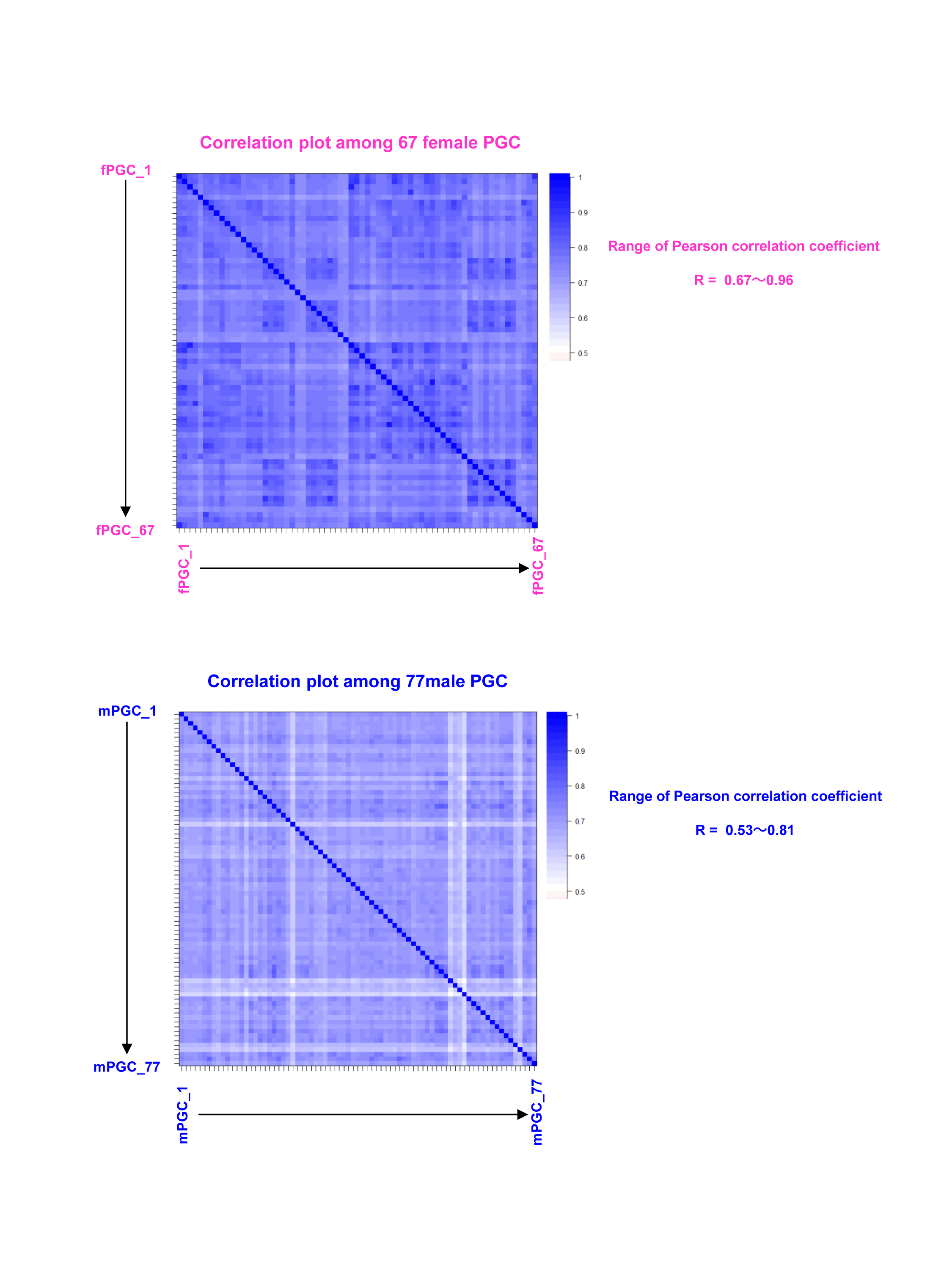

Supplement: S5 Fig — The intensities of the colour gradients indicate the correlation coefficient values between 2 samples. (TIF) [file pone.0144836.s005.tif]

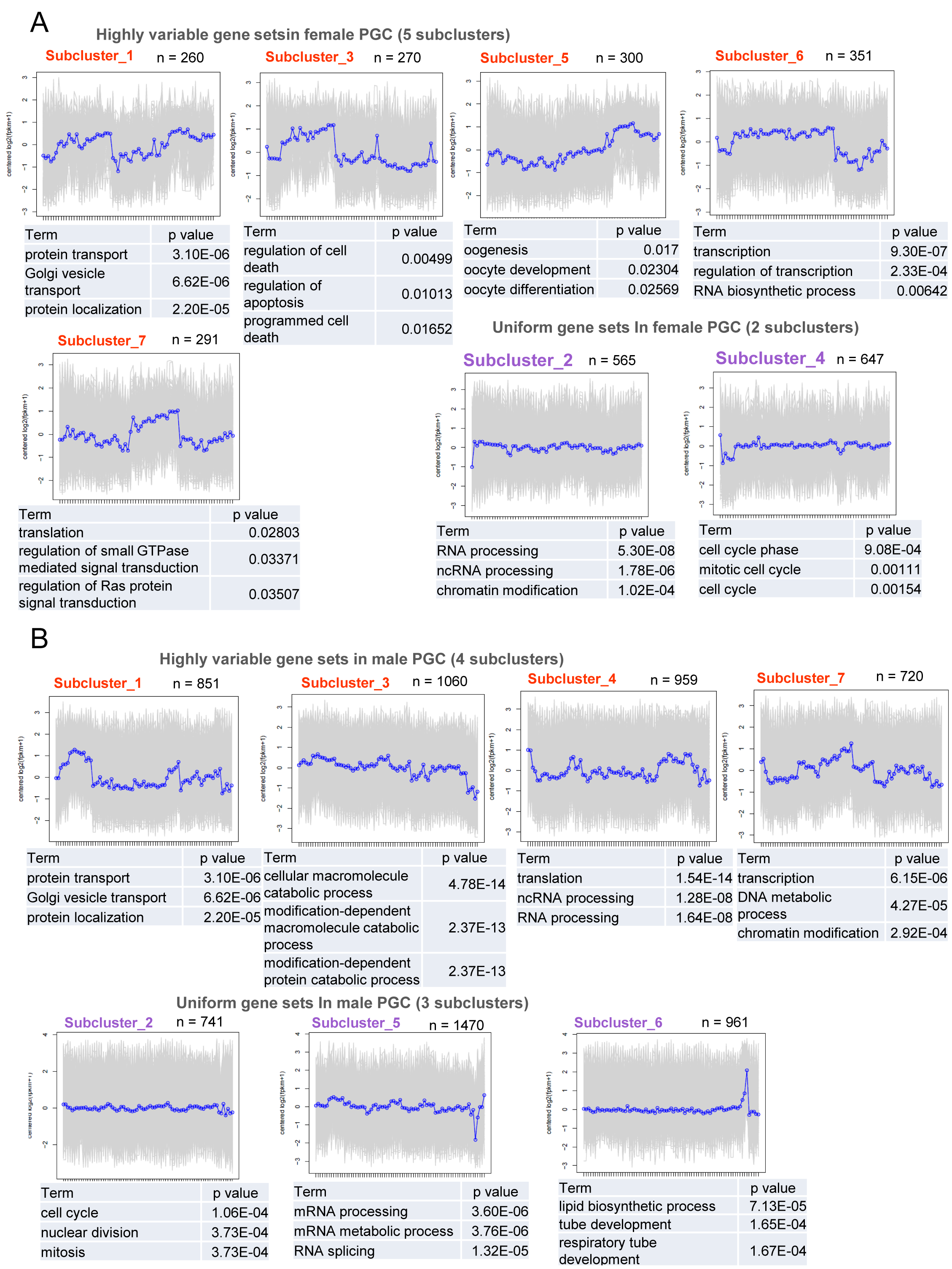

Supplement: S6 Fig — Seven distinct patterns were classified, and the number of genes included in each subcluster is indicated. Expression levels are shown in log2 values, and blue lines indicate the cluster centroid for each subcluster. The most enriched biological processes based on their respective p values are shown (Fisher’s exact test: cut-off < 0.1). (A) female PGCs, (B) male PGCs. (TIF) [file pone.0144836.s006.tif]

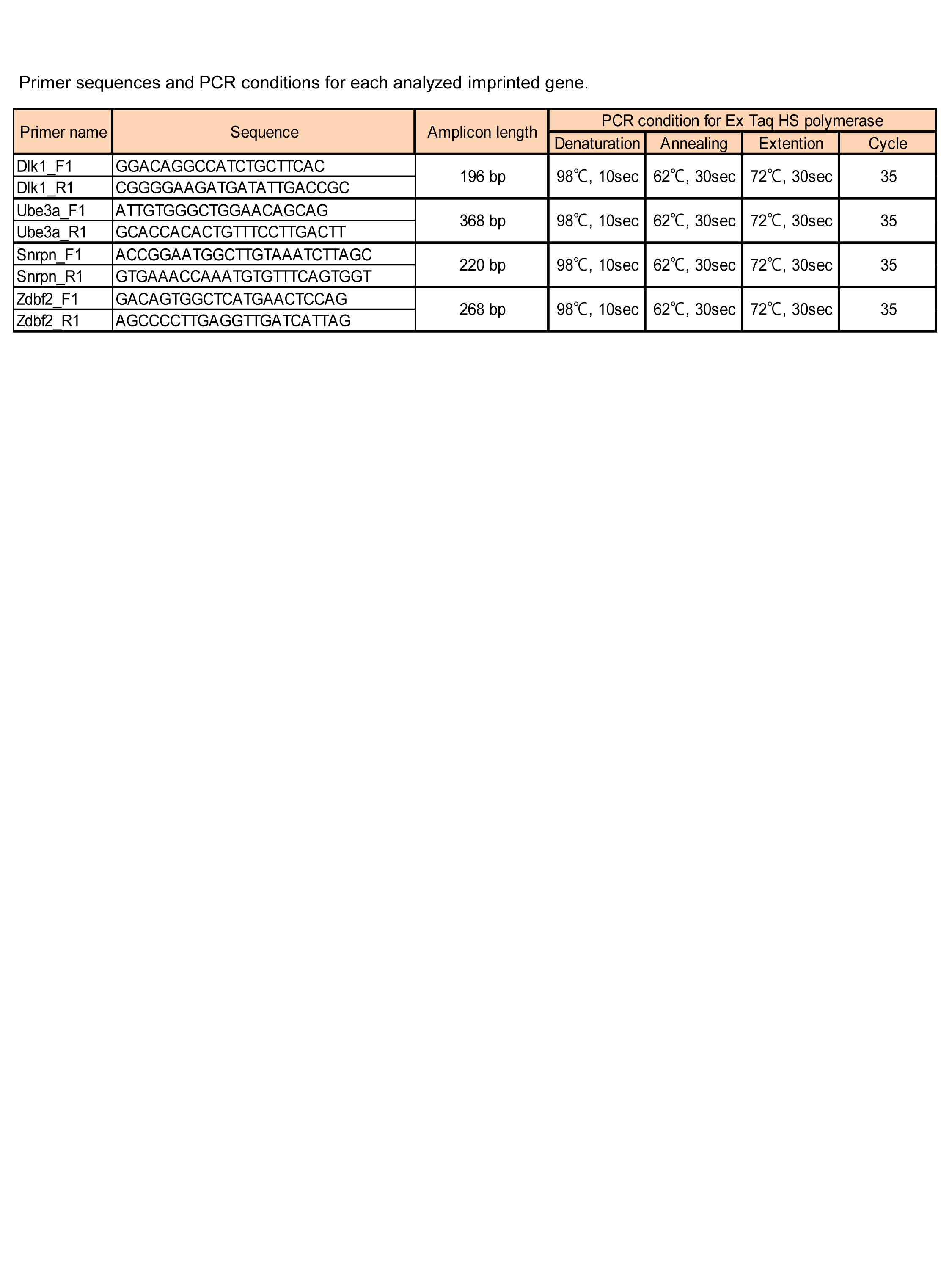

Supplement: S4 Table — (TIF) [file pone.0144836.s010.tif]
